# Workplace bullying among medical laboratory professionals in Ghana: insights from self-reported experiences, challenges to mitigation structures, and coping strategies

**DOI:** 10.1186/s12913-025-12458-6

**Published:** 2025-02-25

**Authors:** Evans Duah, Richard Kobina Dadzie Ephraim, Gabriel Pezahso Kotam, Samuel Mawuli Kumordzi, Samuel Amoah, Nii Armah Addy, Solomon Dzidzornu Yao Kwashie, Abu Abudu Rahamani

**Affiliations:** 1 Clinical Laboratory Department, Cape Coast Teaching Hospital, Cape Coast, Ghana; 2 College of Health and Allied Sciences, School of Allied Health Sciences, Department of Medical Laboratory Science, University of Cape Coast, Cape Coast, Ghana; 3 Central Laboratory, Korle Bu Teaching Hospital, Accra, Ghana; 4 Clinical Laboratory Department, University of Cape Coast Hospital, Cape Coast, Ghana; 5 Institute of Leadership and Management in Education (InLaME), Accra, Ghana; 6 Department of Medical Laboratory Science, University for Development Studies, Tamale, Ghana

**Keywords:** Workplace bullying, Medical laboratory, Ghana, Healthcare productivity, Occupational stress, Human resource

## Abstract

**Background:**

Workplace bullying is a major concern in Ghana’s healthcare sector, often arising from power imbalances and an excessive emphasis on achieving results at the cost of employee well-being. While bullying among healthcare professionals like doctors and nurses is well-documented, little is known about its prevalence among medical laboratory professionals, who play a vital role in patient care. We assessed bullying in this group to inform strategies for mitigation.

**Methods:**

We conducted a cross-sectional survey involving 378 medical laboratory professionals. The survey included demographic information, workplace characteristics, the Revised Negative Acts Questionnaire (NAQ-R), and questions about bullying perpetrators, mental health breaks, and anti-bullying policies. Data analysis involved descriptive statistics, the Kruskal-Wallis test, and logistic regression. Results were reported as frequencies, percentages, and odds ratios with 95% confidence intervals (CIs), and statistical significance set at *p* < 0.05.

**Results:**

44% of the medical laboratory professionals reported experiencing bullying; 29% faced frequent bullying, while 71% encountered it occasionally. Common issues included ignored opinions, unmanageable workloads, gossip, and exclusion. Non-clinical administrative managers were the most frequent perpetrators. Diploma and bachelor’s degree holders had higher odds of being bullied compared to master’s degree holders (AOR = 6.13, *p* = 0.013; AOR = 2.56, *p* = 0.007). Rural professionals had higher odds than urban counterparts (AOR = 2.23, *p* = 0.007).

**Conclusion:**

The high prevalence of workplace bullying among medical laboratory professionals highlights the need for effective policies to enhance workplace conditions and patient care.

**Supplementary Information:**

The online version contains supplementary material available at 10.1186/s12913-025-12458-6.

## Introduction

Bullying is a pervasive global issue that significantly impacts the physical and emotional well-being of its victims. It is often characterized by persistent, unjust behavior including verbal abuse, exclusion, offensive comments, spreading rumors and gossip, threats of violence, and physical abuse, sexual violence, among others [1, 2]. This negative behavior frequently infiltrates institutions and work environments where poor organizational culture prioritizes performance over people [3]. Globally, approximately 23% of individuals have reported being victims of workplace bullying [1]. This behavior is typically driven by power dynamics or the misuse of authority [4] and can lead to low productivity, physical injury, psychological harm, developmental issues, or even death [5–7].

In healthcare, between 61.9% and 78.9% of professionals worldwide have reported experiencing workplace bullying [8, 9], with the prevalence of verbal abuse, threats, physical abuse, and sexual harassment at 57.6%, 33.2%, 24.4%, and 12.4%, respectively [8]. In Africa, workplace bullying has been reported by 9–100% of health professionals [10]. In Ghana, workplace bullying is a significant issue within public health institutions, affecting between 19.1% and 82% of individuals, and is commonly reported among nurses, doctors, allied health professionals, and facility support staff [11–13]. Specifically, there is a paucity of literature on workplace bullying among medical laboratory professionals globally and in most jurisdictions. However, a cross-sectional study conducted in the United States of America (USA) reported that over 68.5% of medical laboratory professionals are victims of workplace bullying [14].

Medical laboratory professionals in Ghana play a critical role in providing essential diagnostic services that support patient care, disease management, treatment, and prognostic decisions [15]. They provide critical diagnostic data that informs approximately 70% of medical decisions [16, 17]. However, several factors make them particularly vulnerable to workplace bullying. Often working behind the scenes, they may receive less visibility and acknowledgment compared to frontline healthcare professionals, leading to diminished recognition of their contributions [18, 19]. Isolation in laboratory environments and limited direct interaction with other healthcare professionals can further amplify feelings of exclusion and vulnerability. On the other hand, hierarchical position within healthcare settings can expose them to power imbalances and exploitation [20]. Additionally, the high-pressure workload, coupled with systemic challenges such as understaffing and inadequate resources, increases stress levels and workplace conflicts [21]. Bullying in this context can significantly affect their mental and physical health, leading to increased stress, anxiety, and burnout. These can impair their focus, reduce job satisfaction, and compromise diagnostic accuracy, directly affecting service quality and patient outcomes [22]. Moreover, a toxic work environment resulting from bullying undermines staff morale and cohesion, contributing to higher turnover rates and staffing shortages [23, 24].

Given the limited literature and prevailing rumors, it is essential to explore the bullying experiences of medical laboratory professionals in Ghana. To the best of our knowledge, this is the first study in Ghana exploring workplace bullying among medical laboratory professionals. This study aims to provide a comprehensive overview of bullying prevalence across various healthcare levels, identify common perpetrators, infer the potential impact on patient care and health facilities, and examine existing coping strategies and organizational measures to address and mitigate such behavior.

## Materials and methods

### Study design

A cross-sectional survey was designed to explore workplace bullying targeting medical laboratory professionals in Ghana. The survey was conducted between January 2024 and August 2024.

### Study participants

The survey was available to the medical laboratory community in Ghana during this period. This community comprises actively practicing medical laboratory professionals in public and private health institutions, and members in research laboratories and academia as enlisted by the Ghana Association of Medical Laboratory Scientists (GAMLS), the professional body of medical laboratory professionals in Ghana. Medical laboratory professionals are essential healthcare workers who perform laboratory tests and analyze biological samples to support diagnosis, treatment, and disease prevention decisions [16, 17]. Their work underpins medical decisions across various disciplines, ensuring accurate and timely results critical for patient care.

### Sample size determination and sampling strategy

At the onset of this survey, the GAMLS registry documented 6,500 registered medical laboratory professionals. A total of 378 medical laboratory professionals were included in the survey, with the sample size determined using the Yamane formula based on the known population size of the target professionals and accounting for a 5% margin of error.

$$\:\text{n}\ge\:\frac{\text{N}}{1+\text{N}\left({\text{e}}^{2}\right)}$$

n = Sample size.

N = Population size (6500).

e = Margin of error (precision level) (0.05).

To optimize survey inclusion and participation, the study utilized a snowball sampling technique. Participants were encouraged to disseminate the survey invitation containing a link to a Google form, which was distributed via email and WhatsApp, to their professional networks and colleagues. All medical laboratory professionals enlisted by GAMLS were eligible to participate in the study, however, retired professionals were excluded.

### Data collection instrument and procedure

We employed a self-administered questionnaire (Supplementary file 1), hosted on Google Forms, which consisted of four sections. These sections were developed based on existing literature and adapted from validated instruments used in similar surveys investigating workplace bullying. The first section collected demographic information of the survey respondents, including biological sex (binary), age in years, highest level of education, region of hometown, tribe or ethnicity, and disability status.

The second section asked questions about workplace characteristics, such as the geographical area where they work, region where the health facility resides, laboratory setting, primary laboratory department, medical laboratory professional’s employment characteristics such as professional cadre, total work experience in years, years spent in private practice, current grade, years of working under current grade and specific role in the medical laboratory (Supplementary file 1).

The third section of the survey used the validated Revised Negative Acts Questionnaire (NAQ-R) [25] to assess workplace bullying among medical laboratory professionals. The NAQ-R, a 22-item tool, evaluates work-related, person-related, and physically intimidating threats based on self-reported experiences over the past 12 months. Responses were scored from 1 (“never”) to 5 (“daily”), with scores above 45 indicating frequent bullying, 33–45 occasional bullying, and below 33 no bullying [14, 25].

The fourth section of the survey instrument contained 16 supplementary questions designed to gather respondents’ self-reported information on workplace bullying (Supplementary file 1). This section sought details about the perpetrators of bullying, the implementation of mental health breaks within the facility, the existence and enforcement of no-bullying policies, reporting structures for bullying victims, and the strategies victims plan to employ to address bullying in the workplace.

Survey invitations with a Google Forms link were sent to medical laboratory professionals via email and WhatsApp using the GAMLS registry. The Google Forms survey was programmed to accept only one response per participant, utilizing email validation to ensure this restriction. Additionally, we included an age verification question requiring respondents to specify their age. Logic was applied to terminate or redirect responses from those who did not meet the age requirement, particularly retirees (above 60 years), to ensure the survey targeted the intended population.

### Variables

#### Outcome variable

The main outcome was the prevalence of workplace bullying among the medical laboratory professionals, calculated using the NAQ-R based on self-reported experiences over the past 12 months. Responses were rated on a scale from 1 (“never”) to 5 (“daily”), with total scores above 45 indicating frequent bullying, scores between 33 and 45 reflecting occasional bullying, and scores below 33 indicating no bullying.

#### Predictor variables

The predictor variables included socio-demographic factors (age in years, sex as a binary variable, highest education level, disability status, and home region/family ties), workplace characteristics (geographical area, facility region, and primary department), and employment characteristics (professional cadre, total years of work experience, and current grade).

### Data analysis

Data analysis began with extracting data from Google Forms into Excel for initial verification. The dataset was then imported into Stata 16, where data cleaning addressed missing values, outliers, and coding accuracy. Descriptive statistics summarized demographic, work-related characteristics, and negative behaviors, with categorical variables reported as frequencies and percentages. Unadjusted and adjusted multivariable logistic regression analyses identified factors associated with workplace bullying, with results reported as odds ratios (ORs) and 95% confidence intervals (CIs). Statistical significance was set at *p* < 0.05, and findings were presented in tables and graphs.

## Results

Table 1 summarizes the demographic data for the study participants, showing a majority aged 31–40 years (52.9%) and predominantly male (78.3%). Education levels varied, with most holding a bachelor’s degree (53.7%), followed by a master’s (19.1%), diploma (18.0%), certificate (3.4%), Doctor of Medical Laboratory Science (MLSD) (5.3%), and doctorate (0.5%). Disability was rare, with only one participant reporting any form (0.3%), while the vast majority reported no disability (98.1%). Home region distribution indicated a higher representation from the southern belt (44.4%), followed by the middle belt (38.4%) and the northern belt (17.2%).

**Table 1:** Socio-demographic characteristics

Variables	*N* = 378, No. (%)
Age group (years)	
20–30	122(32.3)
31–40	200(52.9)
41–50	49(12.9)
51–60	7(1.9)
**Sex (binary)**	
Male	296(78.3)
Female	82(21.7)
**Highest education status**	
Certificate	13(3.4)
Diploma	68(18.0)
Bachelors (4 years)	203(53.7)
MLSD (6 years)^a^	20(5.3)
Masters	72(19.1)
Doctorate	2(0.5)
**Disability**	
Yes, mental, physical, or both	1(0.3)
No, none	371(98.1)
Not sure/don’t know	6(1.6)
**Home region (family ties)** ^b^	
Northern belt	65(17.2)
Middle belt	145(38.4)
Southern belt	168(44.4)

^a^Doctor of Medical Laboratory Science

^b^Northern belt: Northern, North East, Savannah, Upper East, and Upper West regions; Middle belt: Ashanti, Ahafo, Bono, Bono East, and Eastern regions; Southern belt: Greater Accra, Central, Oti, Volta, Western, and Western North regions

Table 2 characterizes the work settings and primary departments of the study respondents. Geographically, the study participants predominantly work in health facilities situated in urban areas of Ghana (57.4%), with smaller representations from rural (23.8%) and peri-urban settings (18.8%). Regionally, most of these facilities are in the southern belt of Ghana (53.4%), followed by the middle belt (34.7%) and the northern belt (11.9%). Regarding facility settings, the largest groups work in district hospitals (27.5%) and teaching hospitals (18.3%), with others from the Christian Health Association of Ghana (CHAG) facilities (17.2%), health centers/polyclinics (17.2%), and various private and specialized facilities. Most of the respondents work in all-in-one laboratories (61.6%), with other significant representations in primary laboratory departments such as haematology (11.1%), microbiology (10.3%), and blood banking (7.1%), among others.

**Table 2:** Workplace characteristics (*N* = 378)

Variables	*n*(%)
**Geographical area**	
Rural	90(23.8)
Peri-urban	71(18.8)
Urban	217(57.4)
**Region** ^a^	
Northern belt	45(11.9)
Middle belt	131(34.7)
Southern belt	202(53.4)
**Facility setting**	
CHAG^b^ facility	65(17.2)
District Hospital	104(27.5)
Health centers and Polyclinics	65(17.2)
Quasi-Government	19(5.0)
Private hospital/Clinic	18(4.8)
Private Laboratory	13(3.4)
Quaternary	2(0.5)
Reference Laboratory/Research Laboratory	8(2.1)
Regional Hospital	15(3.9)
Teaching Hospital	69(18.3)
**Primary department**	
All-in-one laboratory	233(61.6)
Biochemistry or Chemistry	21(5.6)
Blood Banking	27(7.1)
Haematology	42(11.1)
Microbiology	39(10.3)
Molecular	6(1.6)
^c^Others	10(2.6)

^a^Northern belt: Northern, North East, Savannah, Upper East, and Upper West regions; Middle belt: Ashanti, Ahafo, Bono, Bono East, and Eastern regions; Southern belt: Greater Accra, Central, Oti, Volta, Western, and Western North regions

^b^CHAG: Christian Health Association of Ghana

^c^Phlebotomy, histopathology and immunology

Table 3 provides an overview of the study respondents’ professional roles, work experience, and current responsibilities. The majority are Medical Laboratory Scientists (76.7%), with fewer practicing as Medical Laboratory Technicians (17.2%) and Medical Laboratory Assistants (5.8%). Most respondents have 1–5 years of overall work experience (40.5%) and no private practice experience (71.7%). Most study respondents are employed at the Medical Laboratory Scientist (28.6%) and Senior Medical Laboratory Scientist (26.9%) levels, with fewer in either higher or lower grades. Most have worked at their current grade for 1–5 years (92.9%). In terms of roles, a significant proportion have no specific designation (42.3%), while others serve as laboratory managers (22.0%), supervisors (15.3%), and quality managers (12.2%), among other roles.

**Table 3:** Employment characteristics (*N* = 378)

Variable	*n*(%)
**Professional cadre**	
Medical Laboratory Scientist	290(76.7)
Medical Laboratory Technician	65(17.2)
Medical Laboratory Assistant	23(6.1)
**Total work experience (years)**	
1–5	153(40.5)
6–10	140(37.0)
11–20	66(17.5)
21+	19(5.0)
**Years spent in private practice**	
None	271(71.7)
1–5	94(24.9)
6+	13(3.4)
**Current grade**	
Chief Medical Laboratory Scientist	2(0.5)
Chief Technical Officer	1(0.3)
Deputy Chief Medical Laboratory Scientist	22(5.8)
Deputy Chief Medical Laboratory Technician	4(1.1)
Principal Medical Laboratory Scientist	36(9.5)
Principal Medical Laboratory Technician	8(2.1)
Principal Medical Laboratory Assistant	1(0.3)
Senior Medical Laboratory Scientist	102(26.9)
Senior Medical Laboratory Technician	34(8.9)
Senior Medical Laboratory Assistant	16(4.2)
Medical Laboratory Scientist	108(28.6)
Medical Laboratory Technician	33(8.7)
Medical Laboratory Assistant	11(2.9)
**Years worked at current grade**	
1–5	351(92.9)
6+	27(7.1)
**Role in the medical laboratory**	
None	160(42.3)
Consultant	2(0.5)
Laboratory manager	83(22.0)
Supervisor	58(15.3)
Unit head	2(0.5)
Quality manager	46(12.2)
Safety officer	12(3.2)
Education coordinator/instructor	15(4.0)

The categories (not bullied, bullied occasionally, and bullied frequently) were carefully crafted from cumulative scores calculated from the responses to the 22-itemized Revised Negative Acts Questionnaire (NAQ-R). The cutoffs were: “not bullied” (< 33), “occasionally bullied” (33–45), and “bullied frequently” (> 45).

**Fig. 1:**
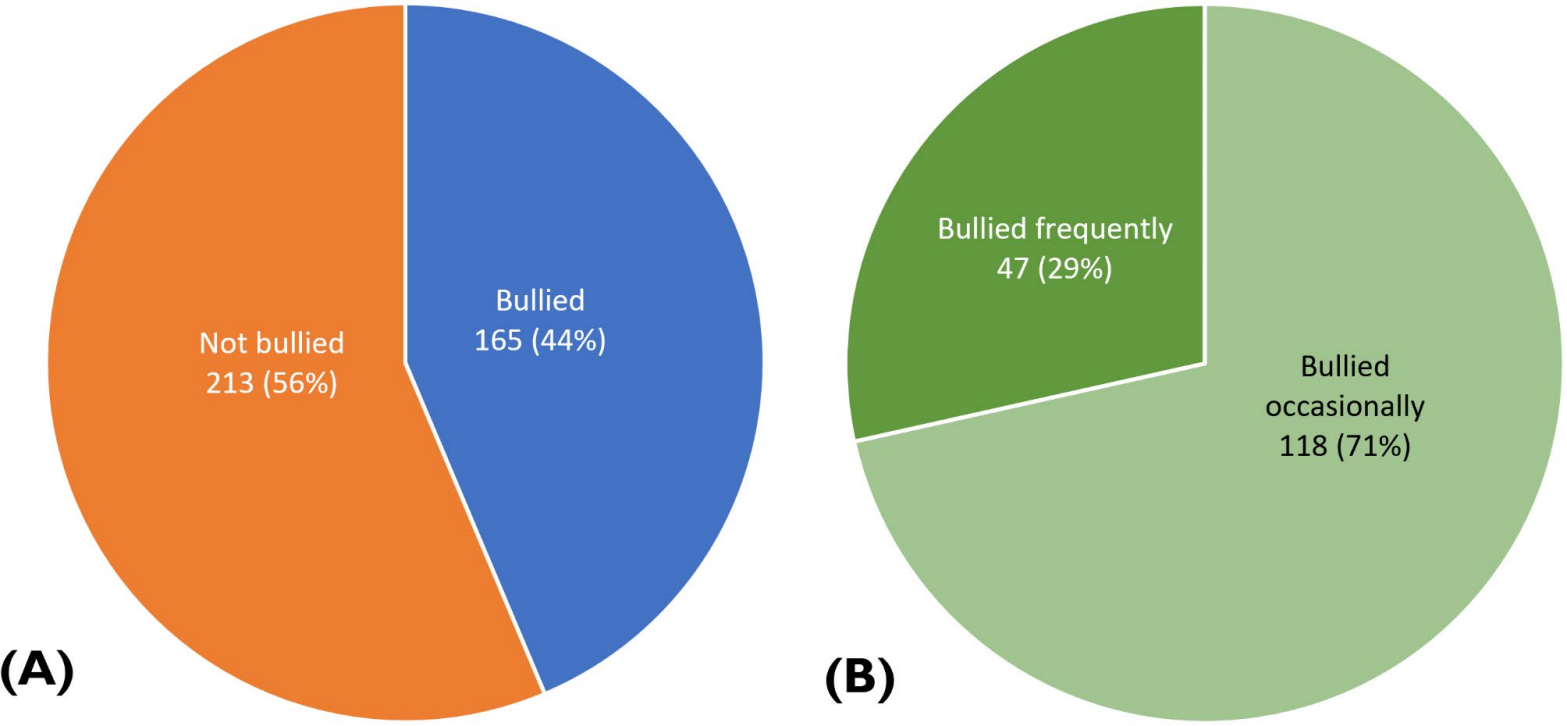
**A** Prevalence of workplace bullying **B** Bullying frequency

Figure 1 provides an overview of the prevalence of workplace bullying among the medical laboratory professionals included in the study. Overall, 165 (44%) respondents reported experiencing bullying (Fig. 1A). Of these, 47 (29%) reported frequent bullying, while 118 (71%) experienced occasional bullying (Fig. 1B).

Table 4 details the frequency of negative behaviors and threats experienced by medical laboratory professionals in the past 12 months, as assessed by the NAQ-R instrument. Respondents reported work-related threats to some degree, including having their opinions ignored (54.5%), being exposed to an unmanageable workload (51.6%), facing pressure not to claim rightful entitlements (46.6%), and experiencing excessive monitoring (40.7%). Weekly or daily occurrences of these threats were reported by 9.8%, 16.4%, 6.9%, and 10.8% of respondents, respectively. Person-related threats, such as spreading gossip (53.7%) and being ignored or excluded (48.1%), were noted, with 9.5% and 5.8% experiencing these issues weekly or daily. Physically intimidating threats were less common, with 26.5% reporting being shouted at and 20.7% encountering intimidating behaviors, while only 2.7% and 3.2% reported these threats occurring weekly or daily.

**Table 4:** Frequency of negative behaviors and threats experienced by the medical laboratory professionals in the past 12 months using the NAQ-R instrument

Threat	No, No. (%)	Yes, to Some degree^a^, No. (%)	Yes, Weekly or Daily^b^, No. (%)
**Work-related threats**			
1. Someone withholding information, which affects your performance	225(59.5)	153(40.5)	28(7.4)
2. Being ordered to do work below your level of competence	260(68.8)	118(31.2)	31(8.2)
3. Having your opinions ignored	172(45.5)	206(54.5)	37(9.8)
4. Being given tasks with unreasonable deadlines	302(79.9)	76(20.1)	13(3.4)
5. Excessive monitoring of your work	224(59.3)	154(40.7)	41(10.8)
6. Pressure not to claim something to which by right you are entitled (e.g., sick leave, holiday entitlement, travel expenses	202(53.4)	176(46.6)	26(6.9)
7. Being exposed to an unmanageable workload	183(48.4)	195(51.6)	62(16.4)
**Person-related threats**			
8. Being humiliated or ridiculed in connection with your work	263(69.6)	115(30.4)	17(4.5)
9. Having key areas of responsibility removed or replaced with more trivial or unpleasant tasks	281(74.3)	97(25.7)	14(3.7)
10. Spreading of gossip and rumors about you	175(46.3)	203(53.7)	36(9.5)
11. Being ignored or excluded	196(51.9)	182(48.1)	22(5.8)
12. Having insulting or offensive remarks made about your person, attitudes, or your private life	242(64.0)	136(36.0)	24(6.4)
13. Hints or signals from others that you should quit your job	270(71.4)	108(28.6)	19(5.0)
14. Repeated reminders of your errors or mistakes	284(75.1)	94(24.9)	15(4.0)
15. Being ignored or facing a hostile reaction when you approach	267(70.6)	111(29.4)	17(4.5)
16. Persistent criticism of your errors or mistakes	294(77.8)	84(22.2)	12(3.2)
17. Practical jokes carried out by people you don’t get along with	290(76.7)	88(23.3)	15(3.9)
18. Having allegations made against you	221(58.5)	157(41.5)	18(4.7)
19. Being the subject of excessive teasing and sarcasm	306(81.0)	72(19.0)	11(2.9)
**Physically intimidating threats**			
20. Being shouted at or being the target of spontaneous anger	278(73.5)	100(26.5)	10(2.7)
21. Intimidating behaviors such as finger-pointing, invasion of personal space, shoving, blocking your way	301(79.3)	77(20.7)	12(3.2)
22. Threats of violence or physical abuse or actual abuse	342(90.5)	36(9.5)	4(1.1)

^a^Includes all categories of yes (now and then, monthly, weekly, and daily)

^b^Includes only categories yes, weekly, and yes, daily

**Fig. 2:**
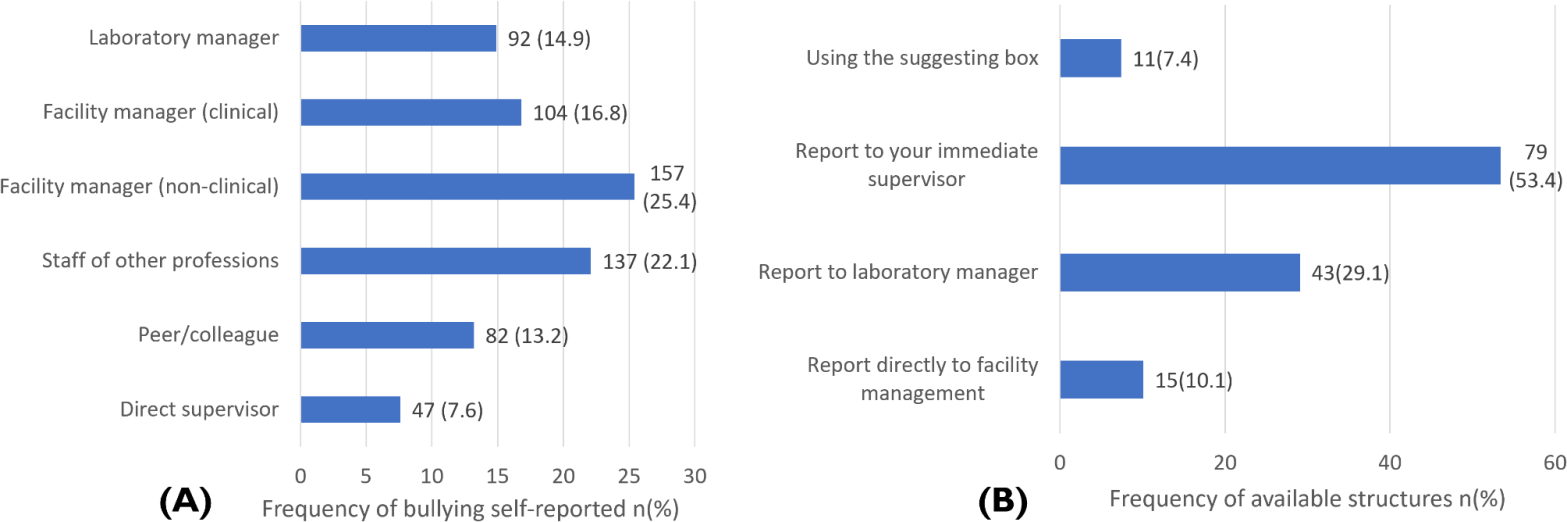
** A** Perpetrators of workplace bullying **B** Structures for reporting bullies.

Figure 2(A) outlines the perpetrators of workplace bullying as reported by the study respondents. Managers responsible for non-clinical administrative tasks were identified as the highest perpetrators of workplace bullying (25.4%), followed by staff from other professions (22.1%), facility managers responsible for clinical decisions (16.8%), laboratory managers (14.9%), colleague medical laboratory professionals (13.2%), and direct supervisors (7.6%).

Figure 2(B) illustrates the structures for reporting workplace bullying as reported by the study respondents. The most common reporting structure was to immediate supervisors, used by 53.4% of the respondents. This was followed by reporting to laboratory managers (29.1%), directly to facility management (10.1%), and using the suggestion box (7.4%).

Table 5 outlines the reasons for workplace bullying as reported by 32 respondents who admitted to bullying others. The most common reason identified was that the bullied individuals frequently made mistakes while working (37.5%). Other notable factors included work-related stress (21.9%) and, a hostile work environment with hateful people (18.8%). Some respondents attributed the bullying to a poor management culture (9.4%). Additionally, a few participants felt compelled to reciprocate or defend themselves due to the prevalent bullying in the facility (6.2%), while others mentioned a desire to demonstrate dominance (6.2%).

**Table 5:** Reasons given by study respondents for bullying others (*N* = 32)

Reason	*n*(%)
Due to poor management culture	3(9.4)
The person always makes mistakes on the bench or when working	12(37.5)
The work environment is full of hateful people	6(18.8)
This facility is full of bullies hence I must reciprocate or defend myself	2(6.2)
To show dominance	2(6.2)
I was stressed at work	7(21.9)

**Fig. 3:**
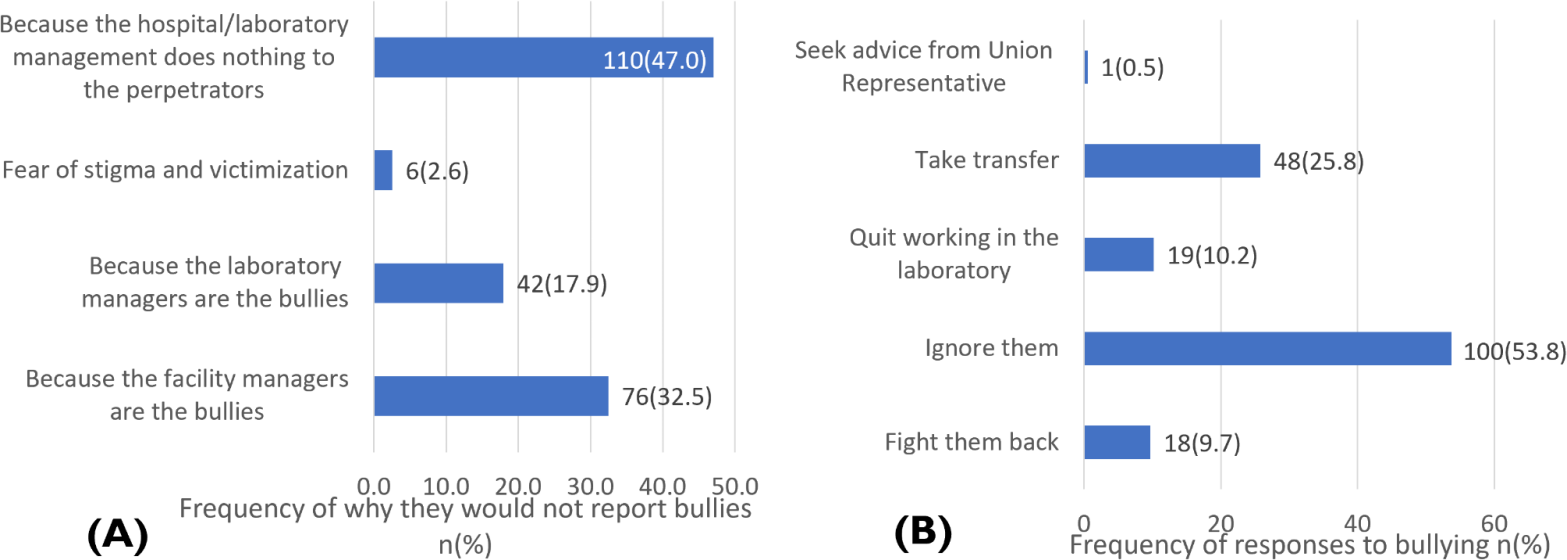
**A** Why they would not report bullies **B** Coping mechanisms

Figure 3(A) presents the reasons some medical laboratory professionals choose not to report incidents of workplace bullying. The predominant reason, cited by 47% of respondents, is that hospital or laboratory managers do not act on such reports. Additionally, 32.5% of respondents refrain from reporting because the facility managers themselves are bullies. Another 17.9% reported that their laboratory managers are the sources of bullying. Fear of stigmatization and victimization was the least common reason for not reporting, cited by only 2.6% of respondents.

Figure 3(B) illustrates how victims of workplace bullying currently address or would prefer to address the issue. Most victims ignore the bullies (53.8%), while others prefer to seek job transfer to different facilities (25.8%). Additionally, 10.2% of respondents would rather quit the profession and pursue other careers, and 9.7% opt to confront the bullies directly.

Table 6 gives the factors associated with workplace bullying among the study participants. The adjusted logistic regression analysis from this study identified significant associations between certain factors and the occurrence of workplace bullying. Specifically, individuals with a diploma had more than six times the odds of experiencing workplace bullying than those with a master’s degree (AOR = 6.13, 95% CI: 1.46–25.68, *p* = 0.013). Similarly, those with a bachelor’s degree had more than twice the odds of being bullied than master’s degree holders (AOR = 2.56, 95% CI: 1.30–5.05, *p* = 0.007). Additionally, professionals working in rural health facilities had over twice the odds of encountering workplace bullying compared to those in urban areas (AOR = 2.23, 95% CI: 1.25–4.00, *p* = 0.007). In contrast, factors such as age, sex, home region, professional cadre, and work experience did not significantly alter the odds of experiencing workplace bullying after adjusting for potential confounders, as indicated by the 95% confidence intervals and p-values greater than 0.05.

**Table 6:** Multivariable logistic regression analysis illustrating factors associated with workplace bullying among the study participants

Variables	Unadjusted odds ratio		Adjusted odds ratio	
	UOR (95%CI)	*P*-value	AOR (95%CI)^a^	*P*-value
**Age group (years)**				
20–30	Reference		Reference	
31–40	0.70(0.44–1.10)	0.125	0.83(0.46–1.51)	0.549
41–50	0.80(0.41–1.57)	0.524	1.06(0.35–3.15)	0.922
51–60	0.78(0.17–3.61)	0.745	1.64(0.16–16.55)	0.677
**Sex (binary)**				
Male	Reference		Reference	
Female	0.97(0.59–1.60)	0.915	0.85(0.49–1.49)	0.577
**Highest education status**				
Certificate	1.13(0.31–4.10)	0.849	0.37(0.03–3.92)	0.407
Diploma	3.87(1.90–7.87)	< 0.001*	6.13(1.46–25.68)	0.013*
Bachelors	2.24(1.25–4.03)	0.007*	2.56(1.30–5.05)	0.007*
MLSD	0.64(0.19–2.14)	0.466	0.56(0.16–2.02)	0.377
Masters	Reference		Reference	
**Home region (family ties)**				
Northern belt	1.39(0.78–2.48)	0.265	1.69(0.89–3.23)	0.110
Middle belt	0.90(0.57–1.41)	0.633	0.91(0.55–1.49)	0.703
Southern belt	Reference		Reference	
**Geographical location of health facility**				
Rural	2.43(1.47–4.03)	0.001*	2.23(1.25–4.00)	0.007*
Peri-urban	1.73(1.01–2.98)	0.048*	1.52(0.84–2.73)	0.165
Urban	Reference		Reference	
**Professional cadre**				
Medical Laboratory Scientist	Reference		Reference	
Medical Laboratory Technician	1.68(0.98–2.89)	0.060	0.47(0.14–1.62)	0.233
Medical Laboratory Assistant	1.44(0.60–3.43)	0.410	2.00(0.31–12.75)	0.465
**Total work experience (years)**				
1–5	Reference		Reference	
6–10	0.61(0.38–0.97)	0.037*	0.77(0.42–1.39)	0.380
11–20	0.70(0.39–1.26)	0.236	1.31(0.52–3.33)	0.565
21+	0.63(0.23–1.71)	0.362	1.16(0.21–6.57)	0.864

^a^adjusted for age, sex, home region, geographical location of health facility, professional cadre, and total work experience

*Statistically significant at *P*-value < 0.05

## Discussion

Our study provides an overview of the prevalence of bullying among medical laboratory professionals across various levels of healthcare in Ghana, identifies common perpetrators, explores the potential impact of workplace bullying, and examines the coping strategies and organizational structures in place to mitigate such behavior.

Nearly 50% of the medical laboratory professionals in this study are exposed to some form of bullying, with the majority encountering it occasionally and a smaller group facing it frequently. This finding aligns with studies reporting a 68.5% prevalence of workplace bullying among medical laboratory professionals in USA [14], 53.1% prevalence among doctors and 53.6% among nurses in Greece [26], 56% prevalence among nurses in Ethiopia [27] and a 38.7% prevalence among anesthetists in South Africa [28]. However, this finding contrasts with studies from Cyprus and the UK, which reported prevalence of 5.9% [2] and 10.6% [29], respectively among healthcare professionals. These discrepancies may arise from differences in geographical and socio-economic contexts. The studies in Cyprus and the United Kingdom were conducted in organizations with more person-centered cultures and working anti-bullying policies compared to the current study. Additionally, most higher-income countries have more advanced systems and structures for monitoring and addressing workplace bullying, unlike Ghana.

In this study, educational attainment and the geographical setting of the health facility were significantly linked to workplace bullying. Specifically, laboratory professionals with a diploma and bachelor’s degree had higher odds of experiencing workplace bullying than those with a master’s degree. These findings align with a similar study that reported healthcare staff with lower levels of education experienced higher rates of bullying compared to those with university-level education [30]. Additionally, in the current study, professionals working in rural health facilities had higher odds of experiencing workplace bullying compared to their urban counterparts. This highlights the unique challenges faced by rural health workers, which include not only limited human and infrastructural resources but also increased exposure to occupational violence and aggression, often perpetrated by community hoodlums [31, 32]. In rural settings of Ghana, limited resources, understaffing, and high workloads can create a tense work environment, increasing the likelihood of bullying. Lower-ranked staff may face power imbalances, discrimination, or mistreatment from supervisors or senior colleagues, further exacerbated by hierarchical workplace cultures. Additionally, isolation in rural facilities, lack of robust anti-bullying policies, and inadequate reporting mechanisms often leave victims with little recourse. These issues do not only affect the mental well-being of professionals but also compromise the quality of diagnostic services, impacting overall healthcare delivery in underserved areas.

Bullies are often perceived as malevolent, exhibiting toxic personality and character traits such as narcissism, self-centeredness, manipulation, deceit, poor anger management, and a tendency toward violence, among other negative behaviors [33, 34]. In many organizations, workplace bullying often stems from power dynamics and the assertion of authority, with managers and those in leadership positions frequently being the perpetrators [4]. In this study, although the perpetrators of workplace bullying vary, the central issue is power imbalance. Health managers responsible for non-clinical administrative tasks were most identified as the main offenders. Other perpetrators included staff from different professions, facility managers involved in clinical decisions, laboratory managers, fellow medical laboratory professionals, and direct supervisors. Similarly, some studies report individuals in leadership positions as the main perpetrators of workplace bullying particularly in healthcare settings. For instance, senior clinical faculty, supervisors, and consultants were identified as the primary perpetrators of workplace bullying in healthcare facilities in Pakistan and Saudi Arabia [35], while senior nurses were the main offenders in bullying newly qualified nurses in a study conducted in South Africa [36]. Additionally, managers within the UK’s National Health Service (NHS) trusts were also reported as significant perpetrators [37].

From this study, it was evident that most health facilities involved had structures in place for reporting and addressing workplace bullying and similar negative behaviors largely through immediate supervisors, laboratory managers, or directly to facility management. However, there are significant challenges with these organizational systems. Many medical laboratory professionals in this study reported reluctance to use these reporting mechanisms, citing issues such as hospital or laboratory managers failing to act on reports, facility and laboratory managers themselves being bullies, and fears of increased victimization and stigma. This mirrors the situation in some UK NHS trusts, where victims of workplace bullying often avoid reporting due to fears of inaction, concerns about being labeled troublemakers, the seniority of perpetrators, and uncertainty about policy enforcement and how the bullying cases would be managed, which can exacerbate their victimization [37]. This phenomenon is similar to what has been observed in some public institutions in Ghana [12].

When organizational systems fail to support professionals, they often take matters into their own hands to address their challenges. In this study, most professionals opted to ignore the bullies as seen in a similar study in South Africa [38] however, a minority chose to confront the bullies more directly. For instance, some medical laboratory professionals admitted to bullying others within their facility, primarily as retaliation in a hostile and toxic work environment or to cope with or resist the prevailing negativity. It was apparent that, influenced by a poor management culture, some professionals felt compelled to either reciprocate or assert dominance as a defense mechanism to avoid being bullied themselves. Attempts to avoid perpetrators have been shown to negatively impact job satisfaction, lead to diminished self-esteem, and adversely affect the psychological well-being of victims [38]. Consequently, those who find the situation intolerable may opt to leave their current positions for transfers to other facilities or even quit the profession entirely to pursue different careers as seen in the current study. This observation aligns with cross-sectional studies in South Korea [39] and South Africa [40], which significantly links workplace bullying to high turnover intentions among both experienced and inexperienced clinical nurses.

## Limitation

This cross-sectional study is limited by its design, which captures data at a single point in time and thus cannot assess temporal changes in workplace bullying or its effects over time. Additionally, the study relies on self-reported data, which may be subject to response bias; participants might underreport or exaggerate their experiences due to social desirability or fear of potential repercussions. While the findings are not intended to be generalized beyond the study sample, they are comparable with those observed in similar study settings and serve as a baseline for further research.

## Conclusion

This study provides valuable insights into the prevalence and impact of workplace bullying among medical laboratory professionals in Ghana, revealing a high prevalence of bullying. Common issues included ignored opinions, unmanageable workloads, gossip, and exclusion, with non-clinical administrative managers as the primary perpetrators of workplace bullying. Lower-ranked and rural professionals were more likely to experience bullying compared to their counterparts. These findings underscore the potential detrimental effects of workplace bullying on job satisfaction and healthcare productivity, ultimately impacting patient outcomes. Addressing these challenges through effective intervention strategies and the implementation of supportive, enforced policies is crucial to improving workplace conditions, enhancing professional well-being, and ensuring quality patient care in Ghanaian healthcare facilities.

## Recommendations

Future studies employing mixed methods could offer a more comprehensive understanding of the bullying phenomenon affecting medical laboratory professionals in Ghana and inform the development and enforcement of no-bullying policies within health facilities.

## Supplementary Information

Supplementary Material 1.

## Data Availability

Data and materials from the study are available upon request from the corresponding author.

## Abbreviations

CHAG: Christian Health Association of Ghana
GAMLS: Ghana Association of Medical Laboratory Scientists
H: Kruskal-Wallis Test Statistic
MLSD: Doctor of Medical Laboratory Science
NAQ-R: Revised Negative Acts Questionnaire
UOR: Unadjusted Odds Ratio
AOR: Adjusted Odds Ratio
UN: United Nations
NHS: National Health Service

## References

[1] United Nations. More than 1 in 5 worldwide suffering from violence at work: ILO. https://news.un.org/en/story/2022/12/1131372 (2022). Accessed 20 Aug 2024.

[2] Zachariadou T, Zannetos S, Chira SE, Gregoriou S, Pavlakis A. Prevalence and forms of workplace bullying among Health-care professionals in Cyprus: Greek version of Leymann inventory of psychological terror instrument. Saf Health Work. 2018;9(3):339–46. 30370167 10.1016/j.shaw.2017.11.003 PMC6129994

[3] Karatuna I, Jönsson S, Muhonen T. Workplace bullying in the nursing profession: A cross-cultural scoping review. Int J Nurs Stud. 2020;111:103628. 32932063 10.1016/j.ijnurstu.2020.103628

[4] De Cieri H, Sheehan C, Donohue R, Shea T, Cooper B. Workplace bullying: an examination of power and perpetrators. Personnel Rev. 2019;48(2):324–41.

[5] Kocakülâh MC, Bryan TG, Lynch S. Effects of absenteeism on company productivity, efficiency, and profitability. Bus Economic Res. 2018;8(1):115–35.

[6] Magnusson Hanson LL, Pentti J, Nordentoft M, Xu T, Rugulies R, Madsen IEH, et al. Association of workplace violence and bullying with later suicide risk: a multicohort study and meta-analysis of published data. Lancet Public Health. 2023;8(7):e494–503. 37393088 10.1016/S2468-2667(23)00096-8

[7] Md S, Md PM, Mahumud RA. Effect of workplace violence on health workers injuries and workplace absenteeism in Bangladesh. Global Health Res Policy. 2023;8(1):33. 10.1186/s41256-023-00316-z PMC10463430 37608337

[8] Liu J, Gan Y, Jiang H, Li L, Dwyer R, Lu K, et al. Prevalence of workplace violence against healthcare workers: a systematic review and meta-analysis. Occup Environ Med. 2019;76(12):927–37. 31611310 10.1136/oemed-2019-105849

[9] Rossi MF, Beccia F, Cittadini F, Amantea C, Aulino G, Santoro PE, et al. Workplace violence against healthcare workers: an umbrella review of systematic reviews and meta-analyses. Public Health. 2023;221:50–9. 37406450 10.1016/j.puhe.2023.05.021

[10] Njaka S, Edeogu OC, Oko CC, Goni MD, Nkadi N. Work place violence (WPV) against healthcare workers in Africa: A systematic review. Heliyon. 2020;6(9):e04800. 32964153 10.1016/j.heliyon.2020.e04800 PMC7490814

[11] Anyomih TTK, Mehta A, Wondoh PM, Mehta A, Siokos A, Adjeso T. Bullying among medical students and doctors in Ghana: a cross-sectional survey. Singapore Medical Journal. 2024. 10.4103/singaporemedj.SMJ-2021-281. 10.4103/singaporemedj.SMJ-2021-281 38779930

[12] Darko G, Björkqvist K, Österman K. Workplace bullying and psychological distress in public institutions in Ghana. Eur J Soc Sci Educ Res. 2019;6(1):62.

[13] Tawiah PA, Appiah-Brempong E, Okyere P, Adu-Fosu G, Ashinyo ME. Prevalence, risk factors and psychological consequences of workplace violence among health workers in the greater Accra region, Ghana: a cross-sectional study. BMC Public Health. 2024;24(1):563. 38388881 10.1186/s12889-024-17962-8 PMC10882733

[14] Chiou PZ, Mulder L, Jia Y. Workplace bullying in pathology and laboratory medicine. Am J Clin Pathol. 2023;159(4):358–66. 36749307 10.1093/ajcp/aqac160

[15] Ephraim RKD, Kotam GP, Duah E, Ghartey FN, Mathebula EM, Mashamba-Thompson TP. Application of medical artificial intelligence technology in sub-Saharan Africa: prospects for medical laboratories. Smart Health. 2024;33:100505.

[16] Duah E, Amoah S, Addy NA, Ephraim RKD. Factors affecting job satisfaction and retention of medical laboratory professionals in Ghana. Afr J Manage Res. 2022;29(1):2–24.

[17] Marinucci F, Majigo M, Wattleworth M, Paterniti AD, Hossain MB, Redfield R. Factors affecting job satisfaction and retention of medical laboratory professionals in seven countries of Sub-Saharan Africa. Hum Resour Health. 2013;11(1):38. 23958152 10.1186/1478-4491-11-38 PMC3751772

[18] Dignos PN, Khan A, Gardiner-Davis M, Papadopoulos A, Nowrouzi-Kia B, Sivanthan M, et al. Hidden and understaffed: exploring Canadian medical laboratory technologists’ pandemic stressors and lessons learned. Healthc (Basel). 2023;11(20):2736. 10.3390/healthcare11202736 PMC10606905 37893810

[19] Jalloh MB, Vernooij E, Street A. Invisible and undervalued: A qualitative study of laboratory workers’ experiences and perceptions of laboratory strengthening in Sierra Leone. Afr J Lab Med. 2024;13(1):11. 10.4102/ajlm.v13i1.2292 PMC11151379 38840958

[20] Fernandopulle N. To what extent does hierarchical leadership affect health care outcomes? Med J Islam Repub Iran. 2021;35:117. 34956963 10.47176/mjiri.35.117 PMC8683790

[21] Kumareswaran S, Muhadi SU, Sathasivam J, Thurairasu V. Prevalence of occupational stress and workload among laboratory staff. Int J Public Health Sci (IJPHS). 2023;12(3):1014–20.

[22] Nowrouzi-Kia B, Dong J, Gohar B, Hoad M. Factors associated with burnout among medical laboratory professionals in Ontario, Canada: an exploratory study during the second wave of the COVID-19 pandemic. Int J Health Plann Manage. 2022;37(4):2183–97. 35306693 10.1002/hpm.3460 PMC9541906

[23] Opoku DA, Ayisi-Boateng NK, Osarfo J, Sulemana A, Mohammed A, Spangenberg K et al. Attrition of Nursing Professionals in Ghana: An Effect of Burnout on Intention to Quit. Behzadifar M, editor. Nursing Research and Practice. 2022;2022:1–9. 10.1155/2022/3100344 PMC9296301 35865623

[24] Wyk SN van, Naicker V. A review of the effect of nurse shortages on existing nurse workforces in South Africa and Ukraine. Technol Audit Prod Reserves. 2023;4(472):28–32.

[25] Einarsen S, Hoel H, Notelaers G. Measuring exposure to bullying and harassment at work: validity, factor structure and psychometric properties of the negative acts Questionnaire-Revised. Work Stress. 2009;23(1):24–44.

[26] Chatziioannidis I, Bascialla FG, Chatzivalsama P, Vouzas F, Mitsiakos G. Prevalence, causes and mental health impact of workplace bullying in the neonatal intensive care unit environment. BMJ Open. 2018;8(2):e018766. 29478015 10.1136/bmjopen-2017-018766 PMC5855440

[27] Bekalu YE, Wudu MA. Prevalence of workplace violence and associated factors against nurses working in public hospitals in Northeastern Ethiopia, 2022. SAGE Open Nurs. 2023;9:23779608231171776. 37250765 10.1177/23779608231171776 PMC10210530

[28] Reddy T, Naidu S. The prevalence and impact of workplace bullying among anaesthetists. South Afr J Anaesth Analgesia. 2024;30(3):79–84.

[29] Bunce A, Hashemi L, Clark C, Stansfeld S, Myers CA, McManus S. Prevalence and nature of workplace bullying and harassment and associations with mental health conditions in England: a cross-sectional probability sample survey. BMC Public Health. 2024;24(1):1–13. 38658961 10.1186/s12889-024-18614-7 PMC11044501

[30] Ariza-Montes A, Muniz NM, Montero-Simó MJ, Araque-Padilla RA. Workplace bullying among healthcare workers. Int J Environ Res Public Health. 2013;10(8):3121–39. 23887621 10.3390/ijerph10083121 PMC3774428

[31] Grant SL, Hartanto S, Sivasubramaniam D, Heritage K. Occupational violence and aggression in urgent and critical care in rural health service settings: A systematic review of mixed studies. Health Soc Care Community. 2022;30(6):e3696–715. 36165419 10.1111/hsc.14039 PMC10086783

[32] Olaniran A, Banke-Thomas A, Bar-Zeev S, Madaj B. Not knowing enough, not having enough, not feeling wanted: challenges of community health workers providing maternal and newborn services in Africa and Asia. PLoS ONE. 2022;17(9):e0274110. 36083978 10.1371/journal.pone.0274110 PMC9462785

[33] Blackwood K, Jenkins M, Me? et al. A Bully? The Different Faces of the Perpetrator in Workplace Bullying. In: D’Cruz P, Noronha E, Baillien E, Catley B, Harlos K, Hogh A, editors. Pathways of Job-related Negative Behaviour. Singapore; 2018. pp. 1–24. 10.1007/978-981-10-6173-8_20-1

[34] Zhang Y, Li Z, Tan Y, Zhang X, Zhao Q, Chen X. The influence of personality traits on school bullying: A moderated mediation model. Front Psychol. 2021;12:650070. 34093338 10.3389/fpsyg.2021.650070 PMC8177084

[35] Ullah R, Siddiqui F, Zafar MS. Bullying among healthcare professionals and students: prevalence and recommendations. J Taibah Univ Med Sci. 2023;18(5):1061–4. 36994224 10.1016/j.jtumed.2023.02.011 PMC10040816

[36] Madolo AN, Hloba SP. Bullying, shortage of staff and resources in workplace: qualitative experience of newly qualified nurses. Curationis. 2023;46(1):1–9. 10.4102/curationis.v46i1.2407 PMC10091067 37042532

[37] Carter M, Thompson N, Crampton P, Morrow G, Burford B, Gray C, et al. Workplace bullying in the UK NHS: a questionnaire and interview study on prevalence, impact and barriers to reporting. BMJ Open. 2013;3(6):e002628.

[38] Bernstein C, Trimm L. The impact of workplace bullying on individual wellbeing: the moderating role of coping. SA J Hum Resource Manage. 2016;14(1):12.

[39] Kim Y, Lee E, Lee H. Correction: association between workplace bullying and burnout, professional quality of life, and turnover intention among clinical nurses. PLoS ONE. 2020;15(1):e0228124. 31945131 10.1371/journal.pone.0228124 PMC6964836

[40] Coetzee M, van Dyk J. Workplace bullying and turnover intention: exploring work engagement as a potential mediator. Psychol Rep. 2018;121(2):375–92. 28812953 10.1177/0033294117725073

