# Supplementary material for: Workplace bullying among medical laboratory professionals in Ghana: insights from self-reported experiences, challenges to mitigation structures, and coping strategies
Source: BMC Health Serv Res. 2025 Feb 25;25:310. doi: 10.1186/s12913-025-12458-6 (PMC11853304; doi:10.1186/s12913-025-12458-6)
Supplement: Supplementary file 1 — Supplementary Material 1. [file 12913_2025_12458_MOESM1_ESM.pdf]

Survey Instrument

A. Socio-demographic characteristics

1. Sex: Male/Female
2. Age:
3. Highest level of education: Certificate/Diploma/Bachelor's/MLSD/Master's/Doctorate
4. Region of hometown (family ties):
5. Tribe/Ethnicity: Akan/Ewe/Ga/Ga-Dangbe/Bono/Dagomba/Nanumba/Mamprusi/Gonja/Komkombas/others
6. Disability: Yes, mental/Yes, physical/Yes, both physical and mental/No, none/Don't know or not sure

B. Workplace characteristics

1. Geographical area of health facility: Urban/Rural/peri-urban (define)
2. Region of health facility:
3. Laboratory setting: CHPS/Health centers and Polyclinics/District Hospital/Regional Hospital/Reference laboratory or Research Laboratory/Teaching Hospital/Quaternary /Quasi-Government /Private hospital/ Private Laboratory/other
4. Primary Laboratory Department: Microbiology/Cytopathology or cytology or histopathology/Molecular/Haematology/Blood Banking/Biochemistry or chemistry/Immunology/Front desk or reception/Phlebotomy/All-in-one laboratory/other
5. Medical Laboratory Professional's Employment characteristics
6. Role in clinical laboratory: Upper management (eg Director, Regional Medical Laboratory Scientist)/Laboratory manager/supervisor/Education coordinator or instructor/Quality manager/Fellow/Consultant/other/none
7. Profession: Phlebotomist/Medical Laboratory Assistant/Medical Laboratory Technician/Medical Laboratory Scientist/Academician, Lecturer or Researcher
8. Total years of work experience:
9. Current cadre or grade: Medical Laboratory Assistant/ Senior Med Laboratory Assistant/Principal Med Lab Assistant/Medical Laboratory Technician/Senior Medical Laboratory Technician/Principal Medical Laboratory Technician/ Medical Laboratory Scientist/Senior Medical Lab Scientist/Principal Medical Laboratory Scientist/Deputy Chief Medical Laboratory Scientist/Chief Medical Laboratory Scientist/Other
10. Years worked at current cadre or grade:

C. Validated Revised Negative Acts Questionnaire (NAQ-R) in the Past 12 months [22 items on a 5-likert scale) (Answers: Never (1), Yes (previously or now) (2), Yes (monthly) (3), Yes (weekly) (4), Yes (daily) (5)]

Work-related threat

1. Someone withholding information, which affects your performance
2. Being ordered to do work below your level of competence
3. Having your opinions ignored
4. Being given tasks with unreasonable deadlines
5. Excessive monitoring of your work
6. Pressure not to claim something to which by right you are entitled (eg, sick leave, holiday entitlement, travel expenses)
7. Being exposed to an unmanageable workload **Person-related threats**
8. Being humiliated or ridiculed in connection with your work
9. Having key areas of responsibility removed or replaced with more trivial or unpleasant tasks
10. Spreading of gossip and rumors about you
11. Being ignored or excluded
12. Having insulting or offensive remarks made about your person, attitudes, or your private life
13. Hints or signals from others that you should quit your job
14. Repeated reminders of your errors or mistakes
15. Being ignored or facing a hostile reaction when you approach 16. Persistent criticism of your errors or mistakes
17. Practical jokes carried out by people you don't get along with
18. Having allegations made against you
19. Being the subject of excessive teasing and sarcasm **Physically intimidating threats**
20. Being shouted at or being the target of spontaneous anger
21. Intimidating behaviors such as finger-pointing, invasion of personal space, shoving, blocking your way
22. Threats of violence or physical abuse or actual abuse

D. Supplementary Questions

1. Perpetrators of work-place bullying (multiple responses): Facility management Administration-Non-clinical (all levels including district and regional managers)/ Facility Management Clinical (all levels including district and regional managers)/Laboratory manager/Direct supervisor/Peers or colleagues/Staff of other professions (eg: nursing, medical, security etc)/
2. Have you taken your annual leave for the past 12 months? Yes/No
3. How many times have you been granted your annual leave since you started working in the laboratory? (Remember: each year, you are entitled to 1 annual leave):
4. Does your facility give a mental health day break or off day? Yes/No
5. Have you ever bullied someone at the workplace? Yes/No

6. If yes, what were the reasons (multiple responses): The person is a foreigner/ the person belongs to an inferior tribe or ethnic group or a tribe I do not like/the work environment is full of hateful, envies and toxic people /the person was not my friend or belong to my direct cohort of relatable peers/the person always makes mistakes on the bench or when working/the person worked with COVID-19 patients or had COVID-19/the person had a disability/I was stressed at work/poor management culture/to command respect/to gain validation/we were competing/I was grieved or traumatized/religious reasons/I don't like people of the opposite sex/the person refused my proposal to have an affair with me/this facility is full of bullies hence I must reciprocate/other
7. Does the facility/laboratory have a no bully policy? Yes/No
8. Have the facility/laboratory provided resources to prevent bullying? Yes/No
9. Is there reporting structures in place to communicate the negative acts to facility/laboratory management? Yes/No
10. If yes, what are the structures? Using the suggesting box/report to your immediate supervisor/report to lab manager/ report directly to facility management/others
11. Do you feel comfortable reporting issues such as bullying to facility/laboratory management? Yes/No
12. If No, why? (multiple responses): Because the hospital/laboratory management does nothing to the perpetrators/ because the facility managers are the bullies/because the laboratory managers are the bullies/other
13. How do you plan to stop the bullies? Take transfer/Fight them back/quit work/report to the Police/other
